# Evidence against continuous variables driving numerical discrimination in infancy

**DOI:** 10.3389/fpsyg.2015.00923

**Published:** 2015-07-02

**Authors:** Ariel Starr, Elizabeth M. Brannon

**Affiliations:** ^1^Department of Psychology and Neuroscience, Duke University, Durham, NCUSA; ^2^Center for Cognitive Neuroscience, Duke University, Durham, NCUSA; ^3^Department of Psychology, University of Pennsylvania, Philadelphia, PAUSA

**Keywords:** quantity representation, analog magnitudes, contour discrimination, size discrimination, numerical cognition

## Abstract

Over the past decades, abundant evidence has amassed that demonstrates infants’ sensitivity to changes in number. Nonetheless, a prevalent view is that infants are more sensitive to continuous properties of stimulus arrays such as surface area and contour length than they are to numerosity. Very little research, however, has directly addressed infants’ sensitivity to contour. Here we used a change detection paradigm to assess infants’ acuity for the cumulative contour length of an array when the array’s surface area and number were held constant. Seven-month-old infants detected a threefold change in contour length but failed to detect a twofold change. These results, in conjunction with previously published data on numerosity discrimination using the same experimental paradigm, suggest that infants are not more sensitive to changes in contour length compared to changes in numerosity. Consequently, these findings undermine the claim that attention toward contour length is a primary driver of numerical discrimination in infancy.

## Introduction

It is well established that infants are sensitive to numerical information in their environment, beginning just hours after birth (Xu and Spelke, 2000; Brannon et al., 2004; Lipton and Spelke, 2004; Xu et al., 2005; Izard et al., 2009; Libertus and Brannon, 2010). Despite these findings, however, debate remains as to whether infants’ representations of number are secondary to representations of continuous quantities that covary with number, such as surface area and contour length (Piaget, 1952; Clearfield and Mix, 1999, 2001; Mix et al., 2002; Clearfield, 2005; Cordes and Brannon, 2009; Cantrell and Smith, 2013; Libertus et al., 2014). An assumption has been that continuous quantities are more immediately available for perception and are therefore more primitive or earlier developing feature representations. Although infants can discriminate arrays on the basis of number when other dimensions are controlled, a related question is whether number is less salient compared to the continuous properties of an array. When presented with an array of objects, do infants preferentially attend to number, or is number a last resort that infants rely on only when other dimensions cannot be used?

Although a few studies indicate that infants may preferentially attend to surface area rather than number when tracking small numbers of objects (Clearfield and Mix, 2001; Feigenson et al., 2002), other research suggests that infants may be more adept at tracking number compared to total surface area, particularly when presented with large numbers of objects (Cordes and Brannon, 2011; Libertus et al., 2014). Cordes and Brannon (2011) found that 7-month-old infants required a fourfold difference to detect a change in the cumulative surface area of an array containing multiple elements, whereas only a twofold change in number resulted in dishabituation. Likewise, Libertus et al. (2014) found that when changes in surface area and number were directly pitted against one another in two dynamic image streams, 7-month-old infants preferred to look at the stream that was changing in number rather than the stream that was changing in surface area. This preference for the numerical change was so strong that a threefold change in number had to be paired with a tenfold change in surface area in order for infants to find the two streams equally engaging. Collectively, these findings suggest that number is actually easier to extract than surface area from arrays containing a large number of elements.

A second variable of interest that covaries with number but has received relatively little attention is contour length (sometimes referred to as perimeter). Some researchers have proposed that in studies purporting to show number discrimination, infants are actually responding to changes in contour length (Clearfield and Mix, 1999, 2001; Mix et al., 2002). Indeed, contour length has been shown to influence numerical discrimination performance even in adults (DeWind et al., 2015). A recent review of infant numerical discrimination studies argued that changes in contour length may explain infants’ looking behavior in many studies that ostensibly controlled for continuous variables in order to isolate numerical discrimination (Cantrell and Smith, 2013). Despite clear evidence that infants are sensitive to changes in visual edges and contour length (Karmel, 1969; McCall and Melson, 1970), few studies have directly investigated infants’ acuity for contour discrimination (Clearfield and Mix, 1999, 2001; Cordes and Brannon, 2009). In one study, infants were habituated to arrays containing either two or three squares and then tested with arrays that contained either a familiar number of squares with a novel contour length or a novel number of squares with a familiar contour length (Clearfield and Mix, 1999). Infants dishabituated only to the arrays with the novel contour length, suggesting that the contour length of the arrays was more salient than the number of elements. However, in an attempt to replicate that finding, another research group found that infants dishabituated to both changes in number and to changes in contour length (Cordes and Brannon, 2009). A point to note is that these studies used a paradigm in which infants were habituated to arrays that were constant in both contour length and number and then tested with arrays in which one variable was held constant and the other was varied. Although this method may inform us as to which of two covarying dimensions infants spontaneously attend, it does not provide a metric of acuity for either dimension.

The present study systematically assessed infants’ sensitivity to changes in contour length using the change detection paradigm to provide a metric of acuity (Ross-Sheehy et al., 2003; Libertus and Brannon, 2010; Starr et al., 2013). Due to the geometry of homogenous dot arrays, relatively small changes in cumulative contour length are necessarily coupled with large changes in cumulative surface area. For example, a threefold change in the contour length of a circle is accompanied by a tenfold change in surface area. Therefore, we constructed irregularly shaped stimuli in order to manipulate contour length while holding surface area constant. In Conditions 1A & B, 7-month-old infants were tested with a twofold change in contour length. In Condition 2, 7-month-old infants were tested with a threefold change. These results were compared to previously collected data using the same paradigm that assessed sensitivity to changes in number (Libertus and Brannon, 2010) and surface area (Libertus et al., 2014) to determine in which dimension infants are most sensitive to change.

## Materials and Methods

### Participants

Forty-two 7-month-old infants participated in this study (mean age 6 months 30 days; range 6 months 15 days to 7 months 15 days; 12 female). Twenty-six infants were tested in Condition 1 (16 in Condition 1A and 10 in Condition 1B). Sixteen infants were tested in Condition 2. Data from eight additional infants were excluded due to parent interference (*n* = 2), excessive fussiness (*n* = 3), failure to complete the experiment (*n* = 2), or video equipment malfunction (*n* = 1). Parents of all infants gave written informed consent to a protocol approved by the local Institutional Review Board.

### Design

Infants were seated in front of two peripheral monitors. During each trial, one monitor displayed a stream of images that contained arrays that were constant in contour length while the other monitor displayed arrays that alternated twofold or threefold in contour length.

### Stimuli

**FIGURE 1:**
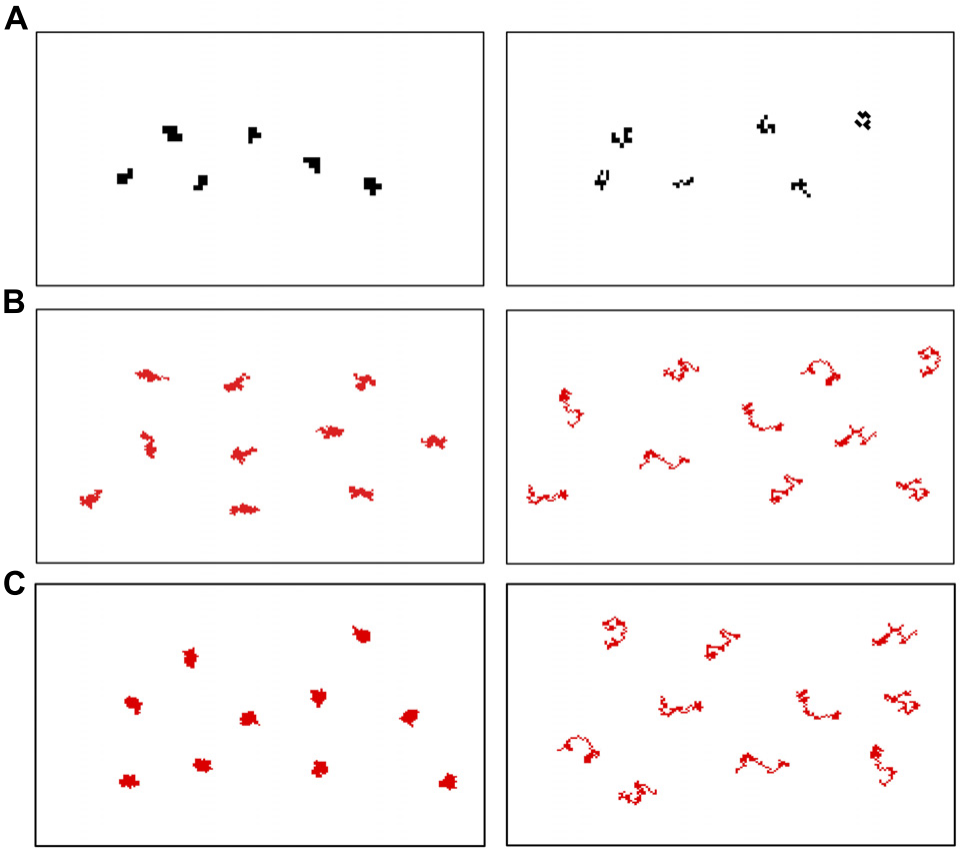
**Example stimuli.** Smaller contour lengths are displayed on the left and larger contour lengths are displayed on the right. **(A)** Condition 1A (twofold change in contour with a 1.5-fold change in area). **(B)** Condition 1B (twofold change in contour with a constant area). **(C)** Condition 2 (threefold change in contour with a constant area).

We used a custom Matlab (Mathworks) program that utilized a random walk technique to create shapes that varied in contour length while holding surface area constant^1^. In Condition 1A, the images in both streams contained arrays of 6 black irregularly shaped items on a white background (see **Figure 1**). The items in each array were constructed with an area of 6 square pixels and a contour length of 12 pixels or an area of 9 square pixels and a contour length of 24 pixels. These shapes were then scaled up to a mean diameter of 2 cm. Note than in Condition 1A, the twofold change in contour length was accompanied by a 1.5-fold change in surface area. Prior research indicates that this change in surface area is below the discrimination threshold for 7-month-old infants (Brannon et al., 2006; Cordes and Brannon, 2008, 2011; Libertus et al., 2014), though it is possible that there could be an additive effect in combination with contour length changes. This concurrent change in surface area was eliminated in Conditions 1B and 2. In Condition 1B, there were 10 (rather than 6) items in each array, and the items in each array were larger in size and red in color to increase their saliency. The items in each array had a constant area of 60 square pixels and contour lengths of either 60 or 120 pixels. The shapes were then scaled up to a mean diameter of 2.5 cm. In Condition 2, the arrays contained arrays of 10 irregularly shaped red items on a white background. The shapes were constructed with a constant area of 60 square pixels and had contour lengths of either 40 or 120 pixels. The shapes were then scaled up to a mean diameter of 1.3 cm. In all conditions, each of the items in a given array had the same contour length but a unique shape. Accordingly, both the contour length of the individual elements and the cumulative contour length of the array varied by a factor of two (Conditions 1A,B) or three (Condition 2) while surface area remained constant (Conditions 1B and 2) or varied by a factor of 1.5 (Condition 1A).

### Procedure

Infants sat in a high chair or on a parent’s lap approximately 105 cm away from three 17-inch monitors. Before each trial, the central screen displayed a colorful attractor video to orient infants’ attention directly ahead. As soon as the infant looked at the attractor stimulus, an experimenter manually started each trial. Each stimulus array was presented for 500 ms followed by 300 ms of black screen. Each stream consisted of four different alternating arrays. Infants were tested with four 60-second trials. The constant and changing streams alternated sides across the four trials for each infant, and the constant stream appeared on the left side first for 50% of the infants. Half of the infants viewed a constant contour stream with arrays that had the longer contour length and the other half of the infants viewed arrays in which the shorter contour length were presented in the constant stream. Infants’ looking behavior was digitally recorded and analyzed oﬄine. An experienced coder blind to the experimental condition coded looking behavior using a custom RealBasic program (Libertus, 2008). A second coder, also blind to the conditions, re-coded 25% of the data and reliability was extremely high across all conditions (*r* = 0.97).

### Data Analysis

Looking time toward each of the visual streams was measured as a percentage of each infant’s total stimulus-directed looking time. Preference scores were calculated by subtracting the proportion of time spent looking at the constant stream from the proportion of time spent looking at the changing stream. A positive preference score therefore indicates a preference for the changing stream, whereas a preference score near zero indicates equal time spent looking at the constant and changing streams.

## Results

**FIGURE 2:**
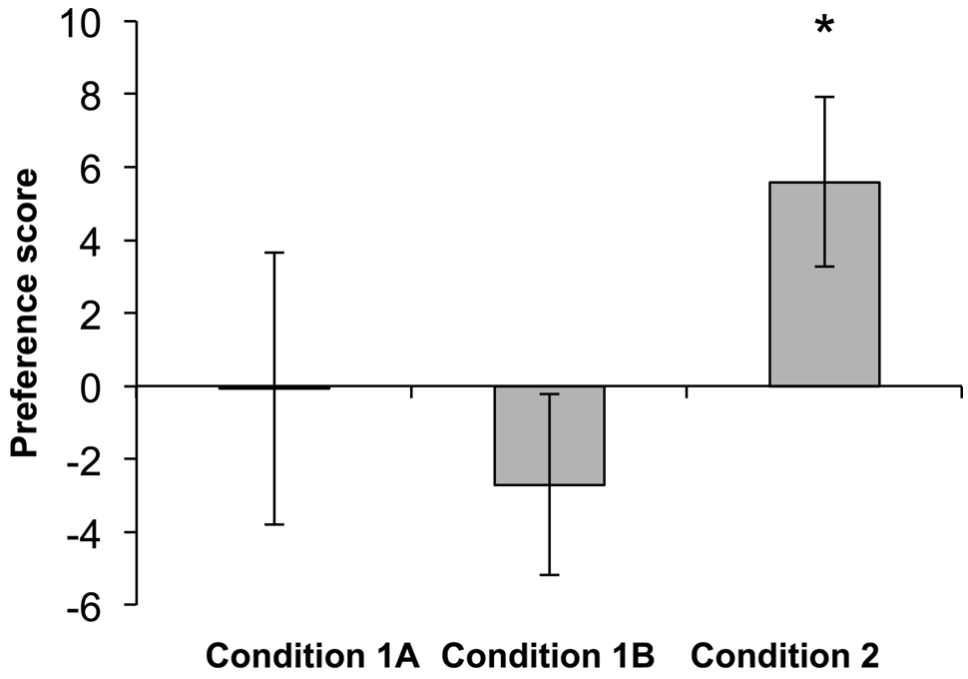
**Results from Experiment 1.** Infants did not exhibit a significant preference for a twofold change in contour length (Conditions 1A,B) but did exhibit a significant preference when the change in contour length was threefold (Condition 2). *p < 0.05.

Preference scores in each condition were analyzed using one-sample *t*-tests comparing the observed preference score to a chance expectation of zero. Infants did not exhibit a preference for the changing contour stream in either Condition 1A [mean preference score = -0.08, *t*(15) = -0.02, *p* = 0.98] or Condition 1B [mean preference score = -2.70, *t*(9) = -01.09, *p* = 0.30]. Furthermore, infants’ preference scores did not differ in Conditions 1A,B [*t*(24) = 0.51, *p* = 0.61]. Thus infants failed to detect a twofold change in cumulative contour length in Condition 1. In Condition 2, however, when contour length in the changing stream differed by a 1:3 ratio, infants exhibited a clear preference for the changing stream (mean preference score = 4.15, *t*(15) = 2.41, *p* < 0.05) (**Figure 2**). Furthermore, a comparison of the mean preference scores from Condition 1 (collapsing across 1A,B) and Condition 2 revealed a trend toward a significantly larger preference score in Condition 2 [*t*(40) = 1.84, *p* = 0.073]. Nonparametric analyses confirm these results. In Conditions 1A,B, only 7 out of 16 and 5 out of 10 infants, respectively, looked longer at the changing contour stream (binomial *ps* > 0.5). By contrast, in Condition 2, 12 out of 16 infants looked longer at the changing contour stream in comparison to the constant contour stream (binomial *p* < 0.05).

**FIGURE 3:**
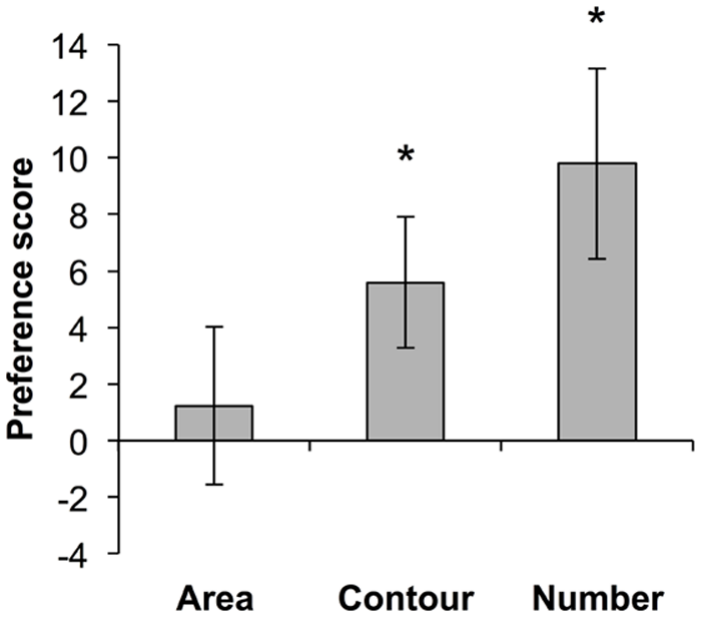
**Comparison of preference scores with a threefold change in cumulative area, cumulative contour, and number (data for area from Libertus et al., 2014; data for number from Libertus and Brannon, 2010).** *p < 0.05.

We next compared infants’ change detection preference scores for a threefold change in cumulative contour (Condition 2) to preference scores from previously published data in which infants of the same age were tested in the same paradigm with threefold changes in cumulative area (Libertus et al., 2014) and number (Libertus and Brannon, 2010). We found a significant linear trend for increasing preference scores, with the lowest scores for cumulative area, intermediate scores for contour length, and the highest scores for numerosity change detection [*F*(1,47) = 4.484, *p* < 0.05] (**Figure 3**). This finding is consistent with previous studies indicating that 7-month-old infants do not detect a threefold change in cumulative area (Cordes and Brannon, 2011; Libertus et al., 2014) but are sensitive to a twofold change in number (Xu and Spelke, 2000; Brannon et al., 2004; Libertus and Brannon, 2010). However, a direct comparison of the mean preference scores for a threefold change in contour compared to a threefold change in number revealed no significant difference between the two dimensions [*t*(30) = 1.03, *p* = 0.313].

## Discussion

In the present study we sought to identify the limits of 7-month-old infants’ ability to detect changes in contour length. To overcome the inherent confound between contour length and surface area present in dot arrays, we created a novel stimulus set that enabled us to vary contour length while holding surface area constant. We then employed these shapes in a change detection paradigm (Ross-Sheehy et al., 2003; Oakes et al., 2006; Libertus and Brannon, 2010). The main result was that infants successfully detected a threefold change in cumulative contour length but were unable to detect a twofold change. Because 7-month-old infants readily detect twofold changes in number (Xu and Spelke, 2000; Brannon et al., 2004; Libertus and Brannon, 2010), this suggests that infants’ acuity for the cumulative contour length in large arrays may be worse than their acuity for number. However, because infants’ require a fourfold change in cumulative surface area in order to recognize change (Cordes and Brannon, 2011; Libertus et al., 2014), it appears that infants are more sensitive to changes in contour length than they are to changes in surface area.

In a few previous studies, it has been reported that infants can detect smaller changes in cumulative contour length, such as 1.5-fold changes, in arrays with two or three items (Clearfield and Mix, 1999, 2001; Cordes and Brannon, 2009). However, there are two critical differences between these prior findings and the results reported here. First, prior studies manipulated contour length in arrays made up of square elements, such that changes in contour length were concurrent with large changes in area. In the present study, we used irregular shapes that enabled us to manipulate contour length while either minimizing changes in surface area or holding surface area constant. It is possible that contour length and surface area have additive effects that facilitate representation and discrimination. If this true, then concurrent change in both area and contour may lead to more precise representations compared to when each dimension is manipulated independently (c.f. Cantrell and Smith, 2013). Note, however, that in Condition 1A, infants were faced with a twofold change in contour length accompanied by a 1.5-fold change in surface area, and there was no evidence that infants detected these concurrent changes. A second difference is that the prior studies employed arrays of two or three elements in habituation paradigms, which may encourage the use of object tracking mechanisms rather than approximate number or approximate magnitude representations (see Feigenson et al., 2004 for a review). Therefore, although object tracking mechanisms may enable more precise representations of contour length, it appears that when many items are present, acuity for contour length, like acuity for surface area, is not as precise as that of number (Cordes and Brannon, 2011; Libertus et al., 2014).

A related topic is the effect of the heterogeneous arrays used in the present study. We employed multiple exemplars of each contour length within the arrays to encourage encoding of contour length rather than specific shapes. Previous work with arrays of one and two items suggests that heterogeneous arrays may encourage infants to attend to numerosity rather than continuous variables such as surface area and contour length when these two variables are pitted against one another (Feigenson, 2005). Although in the present study number was held constant, it is unknown how array heterogeneity influences infants’ attention to number versus continuous properties in arrays containing a larger number of items, which are thought to be represented with analog magnitudes rather than objects tracking mechanisms.

An additional point to consider is that in Conditions 1B and 2, both the cumulative contour length and the contour length of each individual element varied twofold or threefold in the changing stream. Therefore, in theory infants could have attended to either the change in the contour length of an individual element or to the change in the cumulative contour length of the entire array. Prior research, however, suggests that infants do not readily attend to continuous variables of individual elements when presented with large arrays. For example, Cordes and Brannon found that 7-month-old infants required a fourfold change in cumulative surface area of large arrays to detect a change despite readily detecting a twofold change in surface area when presented with a single element (Cordes and Brannon, 2011).

In the present study, changes in contour length were also unavoidably accompanied by a change in shape due to the constraint of holding surface area constant. As a consequence, infants could have attended to changes in either contour length or the overall shape of individual elements. In Condition 1A, we attempted to minimize changes in shape by accompanying the twofold change in contour with a 1.5-fold increase in surface area. In Condition 1B, surface area was held constant which resulted in larger changes in shape. In both of these samples, however, infants did not detect the twofold change in contour length. In addition, the shape changes as quantified by both convex hull and maximum distance were smaller than the changes in contour length. Therefore, it seems unparsimonious to argue that that shape changes accounted for discrimination with a threefold change in contour length in Condition 2 when they were not sufficient to yield discrimination with a twofold change in contour length in Condition 1.

In sum, our results are difficult to reconcile with the idea that numerical information is more difficult for infants to extract compared to information regarding contour length. Our results call into question the proposal that small changes in contour length present in previous studies of infant numerical cognition drive discrimination performance. Instead, it appears that infants are not more sensitive to changes in contour compared to changes in numerosity, which lends credence to the argument that number is indeed a salient dimension for infants.

## Conflict of Interest Statement

The authors declare that the research was conducted in the absence of any commercial or financial relationships that could be construed as a potential conflict of interest.

## Footnotes

1 With the present stimuli set, contour length is the primary determinant of shape, as it is the criteria we have specifically varied while holding other variables constant. In general, however, the gestalt shape of the elements varies across the different contour lengths. Overall, increasing the contour length of an element while holding the area constant results in a more elongated shape, whereas smaller contour lengths produce more compact shapes, as can be seen in **Figure 1**. We have come up with two alternative metrics that could be used to quantify these shapes changes. The first is convex hull, which is defined as the smallest set of convex points that contains each shape. When we compare the average area of these convex hulls, not surprisingly, the area of the convex hulls increases with contour length. However, this increase in convex hull is not proportional to the increase in contour length, and the ratios between the convex hull areas for the three types of images are just 1.02 (larger/smaller) in the twofold condition and 1.03 in the threefold condition. Therefore, it is unlikely that these small changes in convex hull area are driving infants’ looking behavior. A second metric that could be used to quantify shape is the maximum distance between any two points on the perimeter (i.e., overall length). Because the shapes become more elongated as contour length increases, the maximum distance also increases. However, as with convex hull, these differences in maximum distance are smaller than the differences in contour length (a ratio of 1.17 in the twofold condition and a ratio of 2.34 in the threefold condition). Although we believe that contour length is the most parsimonious way to describe the different stimulus types involved in this experiment, these metrics may be helpful for future studies investigating the role of contour length in the formation of quantity representations.

## References

[B1] Brannon E. M. Abbott S. Lutz D. (2004). Number bias for the discrimination of large visual sets in infancy. *Cognition* 93 B59–B68. 10.1016/j.cognition.2004.01.004 15147939

[B2] Brannon E. M. Lutz D. Cordes S. (2006). The development of area discrimination and its implications for number representation in infancy. *Dev. Sci.* 9 F59–F64. 10.1111/j.1467-7687.2006.00530.x 17059447 PMC1661837

[B3] Cantrell L. Smith L. B. (2013). Open questions and a proposal: a critical review of the evidence on infant numerical abilities. *Cognition* 128 331–352. 10.1016/j.cognition.2013.04.008 23748213 PMC3708991

[B4] Clearfield M. W. (2005). “A dynamic account of infant looking behavior in small and large number tasks,” in *Focus on Cognitive Psychology Research*, ed. Vanchevsky M. A. (Hauppauge, NY: Nova Science Publishers), 59–86.

[B5] Clearfield M. W. Mix K. S. (1999). Number versus contour length in infants’ discrimination of small visual sets. *Psychol. Sci.* 10 408–411. 10.1111/1467-9280.00177

[B6] Clearfield M. W. Mix K. S. (2001). Amount versus number: infants’ use of area and contour length to discriminate small sets. *J. Cogn. Dev.* 2 243–260. 10.1207/S15327647JCD0203_1

[B7] Cordes S. Brannon E. M. (2008). The difficulties of representing continuous extent in infancy: using number is just easier. *Child Dev.* 72 476–489. 10.1111/j.1467-8624.2007.01137.x 18366435 PMC2906149

[B8] Cordes S. Brannon E. M. (2009). The relative salience of discrete and continuous quantity in young infants. *Dev. Sci.* 12 453–463. 10.1111/j.1467-7687.2008.00781.x 19371370 PMC2949063

[B9] Cordes S. Brannon E. M. (2011). Attending to one of many: when infants are surprisingly poor at discriminating an item’s size. *Front. Psychol.* 2:65 10.3389/fpsyg.2011.00065 PMC3110486 21687440

[B10] DeWind N. K. Adams G. K. Platt M. L. Brannon E. M. (2015). Modeling the approximate number system to quantify the contribution of visual stimulus features. *Cognition* 132 247–265. 10.1016/j.cognition.2015.05.016 26056747 PMC4831213

[B11] Feigenson L. (2005). A double-dissociation in infants’ representations of object arrays. *Cognition* 95 B37–B48. 10.1016/j.cognition.2004.07.006 15788156

[B12] Feigenson L. Carey S. Spelke E. S. (2002). Infants’ discrimination of number vs. continuous extent. *Cogn. Psychol.* 44 33–66. 10.1006/cogp.2001.0760 11814309

[B13] Feigenson L. Dehaene S. Spelke E. S. (2004). Core systems of number. *Trends Cogn. Sci.* 8 307–314. 10.1016/j.tics.2004.05.002 15242690

[B14] Izard V. Sann C. Spelke E. S. Streri A. Gallistel C. R. (2009). Newborn infants perceive abstract numbers. *Proc. Natl. Acad. Sci. U.S.A.* 106 10382–10385. 10.1073/pnas.0812142106 19520833 PMC2700913

[B15] Karmel B. Z. (1969). Complexity, amounts of contour, and visually dependent behavior in hooded rats, domestic chicks, and human infants. *J. Comp. Physiol. Psychol.* 69 649–657. 10.1037/h0028195 5359138

[B16] Libertus K. (2008). *Preferential Looking Coder.* Available at: http://www.duke.edu/kl41

[B17] Libertus M. E. Brannon E. M. (2010). Stable individual differences in number discrimination in infancy. *Dev. Sci.* 13 900–906. 10.1111/j.1467-7687.2009.00948.x 20977560 PMC2966022

[B18] Libertus M. E. Starr A. Brannon E. M. (2014). Number trumps area for 7-month-old infants. *Dev. Psychol.* 50 108–112. 10.1037/a0032986 23647413 PMC3796133

[B19] Lipton J. Spelke E. S. (2004). Discrimination of large and small numerosities by human infants. *Infancy* 5 271–290. 10.1207/s15327078in0503_2

[B20] McCall R. B. Melson W. H. (1970). Complexity, contour, and area as determinants of attention in infants. *Dev. Psychol.* 3 343–349. 10.1037/h0030032

[B21] Mix K. S. Huttenlocher J. Levine S. C. (2002). Multiple cues for quantification in infancy: is number one of them? *Psychol. Bull.* 128 278–294. 10.1037//0033-2909.128.2.278 11931520

[B22] Oakes L. Ross-Sheehy S. Luck S. (2006). Rapid development of feature binding in visual short-term memory. *Psychol. Sci.* 17 781–787. 10.1111/j.1467-9280.2006.01782.x 16984295

[B23] Piaget J. (1952). *The Child’s Concept of Number.* New York, NY: Norton.

[B24] Ross-Sheehy S. Oakes L. Luck S. (2003). The development of visual short-term memory capacity in infants. *Child Dev.* 74 1807–1822. 10.1046/j.1467-8624.2003.00639.x 14669897

[B25] Starr A. Libertus M. E. Brannon E. M. (2013). Infants show ratio-dependent number discrimination regardless of set size. *Infancy* 18 927–941. 10.1111/infa.12008 PMC3864890 24353478

[B26] Xu F. Spelke E. S. (2000). Large number discrimination in 6-month-old infants. *Cognition* 74 B1–B11. 10.1016/S0010-0277(99)00066-9 10594312

[B27] Xu F. Spelke E. S. Goddard S. (2005). Number sense in human infants. *Dev. Sci.* 8 88–101. 10.1111/j.1467-7687.2005.00395.x 15647069

